# A Rare Cervical Manifestation of Acute Myeloid Leukemia: Granulocytic Sarcoma Simulating as a Gynecologic Tumor

**DOI:** 10.1155/crom/8541641

**Published:** 2025-09-30

**Authors:** Figen Efe Çamili, Gülay Turan, Kübra Bil, Gürhan Güney, Selim Afsar, Mine Islimye Taşkin

**Affiliations:** ^1^Department of Obstetrics and Gynecology, Faculty of Medicine, Balikesir University, Balıkesir, Türkiye; ^2^Department of Medical Pathology, Faculty of Medicine, Balikesir University, Balıkesir, Türkiye

## Abstract

Granulocytic sarcoma (GS) is an extramedullary accumulation of proliferating myeloblasts, most commonly seen in patients with acute myeloid leukemia (AML) but rarely associated with chronic myeloid leukemia, myelodysplastic syndromes, or myeloproliferative neoplasms. The skin is the most frequent site of extramedullary relapse, followed by the mediastinum, gingiva, ear, lymph nodes, central nervous system, and testis. We report a case of a 38-year-old female who presented to our clinic with abnormal vaginal bleeding. She had a history of bone marrow transplantation 5 years ago for AML. Based on clinical suspicion, a cervical biopsy was performed, which confirmed the diagnosis of GS. Although GS involving the female reproductive tract is rare, it should be considered in the differential diagnosis of abnormal gynecologic symptoms in patients with a history of AML.

## 1. Introduction

Granulocytic sarcoma (GS), also known as myeloid sarcoma (MS) or chloroma, is a rare high-grade hematological malignancy characterized by the accumulation of mature or immature myeloid neoplastic cells in extramedullary (EM) sites, which can disrupt the normal tissue structures [1]. According to the fifth edition of the World Health Organization's classification of hematolymphoid tumors, GS can develop as a primary condition or occur in association with acute myeloid leukemia (AML), chronic myeloid leukemia, myelodysplastic disorders, and myeloproliferative neoplasms [2]. It may also serve as an early indication of relapse during the remission phase in AML patients [3]. Studies show that MS accounts for approximately 0.8% of all AML diagnoses [4]. Skin is the most common site for EM relapse, with other frequent locations including the mediastinum, gingiva, ear and external auditory canal, lymph nodes, central nervous system, and testis [1, 3, 5, 6]. These areas can also serve as sites for EM relapse in various conditions. GS of the female reproductive system is rare. When GS affects the female genital tract, the most commonly involved organ is the ovary, followed by the cervix and uterus [7]. We present a case of a 38-year-old female patient who admitted to our clinic with abnormal uterine bleeding and was diagnosed with GS of the cervix as the presenting feature of AML.

## 2. Case Presentation

**Figure 1:**
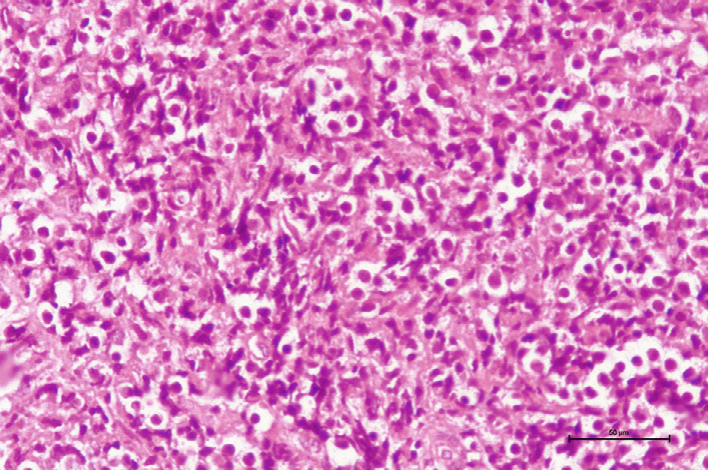
Sheets of medium-sized immature cells with clear cytoplasm and abundant mitotic figures (H&E ×400).

**Figure 2:**
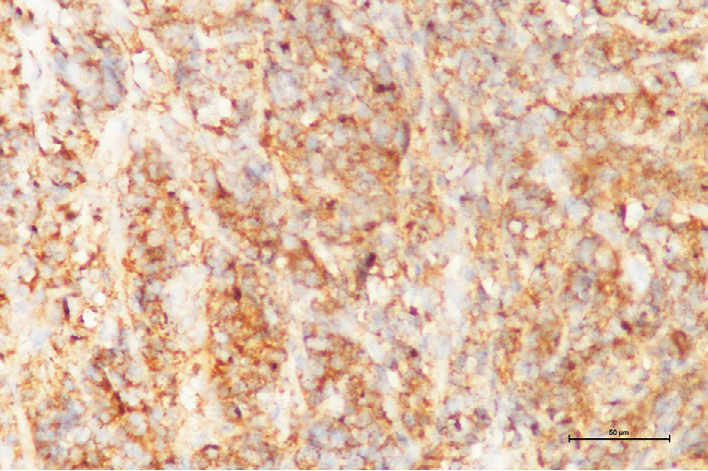
Strong cytoplasmic immunoreactivity for myeloperoxidase (MPO ×400).

A 38-year-old female patient, Gravida 3, Parity 3, with a history of three cesarean deliveries, presented with abnormal vaginal bleeding. She has a known diagnosis of AML and underwent a bone marrow transplant 4 years ago. A transvaginal ultrasound was performed, revealing the presence of an intrauterine device (IUD) in the uterine cavity, a 7.5-cm Type 3 myoma at the fundus, and bilaterally normal ovaries. The speculum examination showed that the cervix was hemorrhagic, and the IUD string was not visible. The patient's complete blood count shows low hemoglobin levels: 11.0 g/dL, normal total leukocyte count: 7.1 × 10^3^/*μ*L, differential leukocyte count within normal limits, and a normal platelet count: 265 × 10^3^/*μ*L. Given the clinical findings, the patient was scheduled for a hysteroscopic IUD removal and laparoscopic myomectomy. The patient's complete blood count shows low hemoglobin levels, normal total leukocyte count, differential leukocyte count within normal limits, and a normal platelet count. However, the patient was admitted to the hospital due to ongoing heavy vaginal bleeding and a significant drop in hemoglobin levels (Hgb 7.2 g/dL). She was transfused with 2 units of red blood cell suspension (RBCs) and received 1 g of tranexamic acid. Despite initial management, the patient continued to experience active bleeding and was taken for emergency surgery. During cervical dilation, suspicion of malignancy arose, leading to a cervical biopsy, as friable, exophytic lesions were observed on the cervix during inspection. Hysteroscopy was subsequently performed, revealing no IUD and a normal uterine cavity. The patient was closely monitored postoperatively until her vaginal bleeding completely ceased. She was transfused with 2 units of RBCs and 1 unit of platelet transfusion and received further tranexamic acid. The patient's hemoglobin improved to 11 g/dL, and she was discharged. The pathology report confirmed the presence of AML infiltration in the cervix, diagnosed as GS. The pathological diagnosis was AML. Histopathological examination revealed sheets of medium-sized immature cells with clear cytoplasm and numerous mitotic figures (Figure 1). Immunohistochemical staining showed strong cytoplasmic positivity for myeloperoxidase (MPO) in these cells (Figure 2), and further immunohistochemical analysis revealed the following characteristics in the neoplastic cells: positive markers: CD56 (+), MPO (+), CD34 (+), CD33 (+), HLADR (+), Bcl2 (+), and CD117 (+); negative markers: TdT (−), Bcl6 (−), MUM1 (−), CD3 (−), CD20 (−), PAX5 (−), and CD10 (−). Additionally, Ki-67 staining demonstrated increased proliferation in the neoplastic cells, indicating a high level of cell division and activity. These findings are consistent with an AML diagnosis, characterized by the presence of myeloid lineage markers (MPO, CD33, CD34, and CD117) and the absence of lymphoid markers (CD3, CD20, PAX5, and CD10). The increased Ki-67 proliferation index further supports the aggressive nature of the disease.

The patient was subsequently referred to the oncology department, where the diagnosis was confirmed by bone marrow aspiration. Further follow-up and treatment have been carried out at another center where a specialized oncology clinic is available.

## 3. Discussion

GS is a rare EM manifestation of AML, typically involving tissues such as the skin, lymph nodes, and soft tissues. We report a unique case of cervical GS representing an isolated EM relapse of AML, diagnosed exclusively due to abnormal uterine bleeding in the absence of a cervical mass. Cervical involvement is exceedingly rare and can present as a mass or mimic other conditions like cervical tumors or fibroids, as seen in this case. In the present case, the patient, who had a known history of AML and had previously undergone allogeneic bone marrow transplantation, presented with sudden and profuse vaginal bleeding. The hemorrhagic appearance of the cervix and the absence of the IUD string initially suggested a gynecologic malignancy, prompting a hysteroscopic evaluation during which a biopsy was performed. Histopathological examination subsequently confirmed the diagnosis of GS, representing a relapse of AML in the form of EM disease. This case highlights the diagnostic complexity of cervical GS, which may present with nonspecific symptoms that overlap with more common gynecologic conditions. In a review of 25 cases by Pathak et al. in 2022, cervical GS was reported to have 83% of patients presenting with abnormal or postcoital bleeding, while others experienced abdominal pain (29%) or systemic symptoms such as fever or fatigue (17%) [8]. Also, in a review of 42 cases by Zhu et al. in 2024, vaginal bleeding was the most common symptom and usually resulted in the detection of cervical masses [9]. Despite the variability in clinical presentations (e.g., abdominal pain, fever, and menstrual irregularities), all cases had a mass that could be detected by imaging or physical examination. However, in our case, the diagnosis was reached only with abnormal bleeding without any cervical mass. These findings emphasize the importance of considering GS in the differential diagnosis of abnormal uterine bleeding, particularly in patients with a history of hematologic malignancy. Early recognition and tissue diagnosis are crucial for prompt management, as delayed diagnosis may impact treatment outcomes and overall prognosis.

In the case series by Li et al. in 2024, it is reported that cervical GS developed as an isolated EM relapse of AML, and the diagnosis was confirmed through histopathological and immunohistochemical evaluations [10]. Immunohistochemical analysis of the 20 patients revealed a high positivity rate for key markers: MPO was positive in all 20 tested cases (100.0%), CD68 was positive in 7 out of 10 cases (70.0%), lysozyme in 8 out of 9 cases (88.9%), CD43 in all 15 tested cases (100.0%), CD117 in 11 out of 15 cases (73.3%), CD34 in 9 out of 16 cases (56.3%), CD15 in 3 out of 9 cases (33.3%), CD99 in 5 out of 7 cases (71.4%), and both CD20 and CD3 were not expressed in any of the 17 and 18 cases tested, respectively. In our case, the presence of myeloid lineage markers (MPO, CD33, CD34, and CD117) confirmed the diagnosis of AML, and the increased Ki-67 proliferation index further supported the aggressive nature of the disease. A similar immunophenotypic profile was observed in our case; however, unlike the patients in Li et al.'s study who predominantly presented with a detectable cervical mass, our patient exhibited only abnormal vaginal bleeding without any identifiable mass lesion. This finding highlights that GS can manifest in a nonmass-forming manner, presenting solely with bleeding symptoms, which represents a deviation from the typical presentations reported in the literature. In the case reported by Weingertner et al. in 2009, cervical GS developed secondarily following thyroid malignancy and presented as a precursor to AML [11]. In contrast, our case involved a patient with a previously diagnosed AML, in whom an isolated EM relapse occurred in the cervix during bone marrow remission. This highlights that EM GS in AML patients can manifest either as an early indicator of disease or as a late relapse occurring years after remission. Therefore, clinicians should remain vigilant for the possibility of EM relapses not only in the initial stages of AML but also long after apparent remission, and long-term follow-up protocols should be adapted accordingly.

The management of GS should indeed focus on treating the underlying systemic disease rather than just the localized tumor. Appropriate chemotherapy, aimed at targeting the AML itself, is essential for improving patient outcomes. The treatment options for GS are indeed multifaceted and depend on the individual patient's condition, the extent of disease, and whether there is an underlying AML or other hematologic malignancies. The options include systemic chemotherapy, local radiotherapy, surgery, and allogeneic or autologous bone marrow transplantation [12].

Prognosis for GS of the cervix, whether or not there is a history of AML, is generally poor, with a 5-year survival rate of approximately 20% [13]. Given the diagnostic complexity and poor prognosis of cervical GS, multidisciplinary collaboration and heightened clinical suspicion are critical for early and accurate diagnosis, especially in patients with a history of AML.

Although several case reports have been published, large-scale studies on the management and outcomes of cervical GS are still lacking. This case highlights the importance of considering GS in the differential diagnosis of patients with AML who present with unusual or unexplained symptoms, such as abnormal bleeding, particularly in areas where EM infiltration is uncommon.

## 4. Conclusion

GS should be considered in the differential diagnosis when a patient with a history of AML presents with unusual symptoms like abnormal bleeding or cervical masses. Early recognition and biopsy are crucial for the timely diagnosis and appropriate management of this rare condition.

## Data Availability

The data that support the findings of this study are available on request from the corresponding author. The data are not publicly available due to privacy or ethical restrictions.
